# What Adolescents Say in Text Messages to Motivate Peer Networks to Access Health Care and Sexually Transmitted Infection Testing: Qualitative Thematic Analysis

**DOI:** 10.2196/44861

**Published:** 2024-02-28

**Authors:** Marguerita Lightfoot, Chadwick Campbell, Allysha C Maragh-Bass, Joi Jackson-Morgan, Kelly Taylor

**Affiliations:** 1 School of Public Health Oregon Health & Science University – Portland State University Portland, OR United States; 2 Division of Prevention Science University of California, San Francisco San Francisco, CA United States; 3 Herbert Wertheim School of Public Health & Human Longevity Science University of California, San Diego San Diego, CA United States; 4 Behavioral, Epidemiological, Clinical Sciences Division FHI 360 Durham, NC United States; 5 Duke Global Health Institute Duke University Durham, NC United States; 6 3rd Street Youth Center and Clinic San Francisco, CA United States; 7 Institute for Global Health Sciences University of California, San Francisco San Francisco, CA United States

**Keywords:** adolescents, clinics, HIV/STI testing, intervention, mobile health, peer, screening, sexually active, STI, text messaging, young adult

## Abstract

**Background:**

While rates of HIV and sexually transmitted infections (STIs) are extremely high among adolescents and young adults in the United States, rates of HIV and STI testing remain low. Given the ubiquity of mobile phones and the saliency of peers for youths, text messaging strategies may successfully promote HIV or STI testing among youths.

**Objective:**

This study aimed to understand the types of messages youths believe were motivating and persuasive when asked to text friends to encourage them to seek HIV or STI testing services at a neighborhood clinic.

**Methods:**

We implemented an adolescent peer-based text messaging intervention to encourage clinic attendance and increase STI and HIV testing among youths (n=100) at an adolescent clinic in San Francisco, California. Participants were asked to send a text message to 5 friends they believed were sexually active to encourage their friends to visit the clinic and receive STI or HIV screening. Thematic analysis was used to analyze the content of the text messages sent and received during the clinic visit. Member checking and consensus coding were used to ensure interrater reliability and significance of themes.

**Results:**

We identified four themes in the messages sent by participants: (1) calls to action to encourage peers to get tested, (2) personalized messages with sender-specific information, (3) clinic information such as location and hours, and (4) self-disclosure of personal clinic experience. We found that nearly all text messages included some combination of 2 or more of these broad themes. We also found that youths were inclined to send messages they created themselves, as opposed to sending the same message to each peer, which they tailored to each individual to whom they were sent. Many (40/100, 40%) received an immediate response to their message, and most participants reported receiving at least 1 positive response, while a few reported that they had received at least 1 negative response. There were some differences in responses depending on the type of message sent.

**Conclusions:**

Given the high rates of STI and HIV and low rates of testing among adolescents, peer-driven text messaging interventions to encourage accessing care may be successful at reaching this population. This study suggests that youths are willing to text message their friends, and there are clear types of messages they develop and use. Future research should use these methods with a large, more diverse sample of youths and young adults for long-term evaluation of care seeking and care retention outcomes to make progress in reducing HIV and STI among adolescents and young adults.

## Introduction

Adolescents are overrepresented among those diagnosed with sexually transmitted infections (STIs). More than half of all STIs in the United States are among adolescents aged between 15 and 24 years [1]. More specifically, adolescents aged between 15 and 24 years comprise 62.6% of all chlamydia infections, and there were increases in rates of gonorrhea, syphilis, and chlamydia incidence among young men and women in this age group between 2016 and 2017 [1]. However, STI testing rates among adolescents are low. Only 27.8% of sexually experienced females and 9.6% of sexually experienced male candidates, aged between 15 and 25 years, reported being tested for STIs in the previous 12 months. Importantly, there are gender differences in testing rates. Among those with 4 or more lifetime partners, 40.8% of females reported receiving an STI test in the previous 12 months, compared to only 3.1% of males [2]. Numerous barriers to STI testing have been identified, including concerns about confidentiality, embarrassment, limits to access (eg, clinic hours and payment), and low-risk perception [1,2].

In 2018, adolescents aged between 13 and 24 years accounted for 21% of new HIV diagnoses in the United States and were the least likely to be virally suppressed of all groups living with HIV [3]. Among those aged between 13 and 19 years, the highest rate of new diagnoses was among African American individuals. Among those aged between 20 and 24 years, those identifying as African American, multiracial, and Hispanic had the first, second, and third highest new diagnosis rates, respectively. These rates are alarming, and they are between 2 and 7 times the rate of new diagnoses for all adults and adolescents in the United States [1]. Despite the high percentage of HIV diagnoses among teens and young adults, similar to STI testing rates, HIV testing rates are low. Among high school students who have ever had sex, the estimated proportion of having ever received an HIV test is between 13% and 22%, and only 33% of those aged between 18 and 24 years have ever tested for HIV [4]. It is estimated that only 40% of those aged between 13 and 29 years who live with HIV are aware of their status [3]. Many barriers exist related to adolescents and young adults getting tested and knowing their status. Some adolescents reported low perceptions of risk, concerns about confidentiality, difficulty remembering to go and get tested, and very few cues to action for HIV testing, such as being offered a test by a medical provider [5]. Profoundly apparent in these numbers, strategies that encourage adolescents and young adults to get tested for STIs and HIV are desperately needed.

A model that uses peer-driven technology-based communication methods to encourage testing among youths may be effective in decreasing the barriers reported in this population. Recent data suggest that, across race and ethnicity, gender, and socioeconomic status, as many as 95% of teens in the United States have access to a smartphone [6]. Text message–based interventions have shown promise in affecting positive changes in health behavior for a number of health issues, including diabetes, weight loss, medication adherence, and smoking cessation [7]. With widespread phone access among American adolescents and with the average youth sending more than 30 messages a day, mobile phone–based health interventions offer an opportunity to reach many adolescents and young adults to improve health outcomes [8]. Importantly, youths have responded positively to receiving SMS text messages as a part of health promotion activities. Youths (aged between 16 and 20 years) who identified as African American and heterosexual expressed openness to receiving HIV prevention and sexual health messages by text and expressed a desire for a text intervention to include the opportunity to build community and share information with each other [9]. A study of adolescents aged between 15 and 20 years described text messaging as convenient for health promotion because they are “always on their phones” [10] and a text-based peer education intervention in which youths could text sexual health questions to a peer health educator was found to be feasible and acceptable [8]. However, in each of these interventions, text messages were sent by clinic or research staff, and the messages themselves were standardized.

Peer strategies are based on the premise that members of a social network know and trust each other and often share similar health behaviors [11]. Building on the widespread usage of text messaging and the important role of peer influence, we implemented a peer-delivered texting intervention to increase attendance at a youth health clinic in San Francisco. This word-of-mouth communication strategy [12] is a practical application of diffusion theory [13]. Studies indicate this communication strategy is highly persuasive [14]. The implementation of the peer-based text messaging strategy showed promise for both increasing the number of youths accessing health services and finding youths engaging in sexual risk behaviors and most in need of sexual health screening and services [15]. This study will examine the SMS text messages that adolescents and young adults sent in order to explicate the types of messages they believed would be persuasive among their friends. Understanding what young people find motivating and persuasive can inform other peer-based models of influence and intervention.

## Methods

### Study Design

We analyzed the content of messages sent and received during the initial clinic visit.

### Study Population

A total of 100 participants were recruited from an adolescent medicine clinic in San Francisco, California, which served youths aged between 12 and 24 years. We approached consecutive adolescent clinic patients to send text messages to 5 peers they believed were sexually active and lived in the clinic’s service area. Of the 153 patients approached by research staff, 18% (28/153) refused, 16% (25/153) did not have a phone with them, and 65% (100/153) provided informed consent and sent text messages to at least 5 peers.

Participants were told that the goal of their messages was to encourage their friends to visit the clinic and receive STI screening. Participants developed their own messages and were provided with an SMS text messaging guide that reviewed considerations for developing messages (eg, let your friend know you care about them, example messages). The SMS text message and any responses from friends while the participant was still in the clinic were recorded by research staff.

### Data Analysis

We analyzed text messages using thematic analysis, as described by Braun and Clarke [16,17]. The second author conducted the initial analysis, including reading all text messages, generating initial codes, and identifying emergent themes. After completing the initial analysis, the first author reviewed the codes and initial themes. Together, the first and second authors discussed the initial analysis, merged related themes, identified the most salient themes, and eliminated those that lacked enough supporting data. Decision trails were maintained throughout the coding and analysis process.

### Ethical Considerations

The study was approved by the University of California, San Francisco, institutional review board (#12-08516).

## Results

### Participants

A total of 430 new patients were seen at the clinic during data collection, of whom 78.6% (338/430) were female. The new patients were 61.2% (263/430) African American, 17.4% (75/430) Hispanic, had a mean age of 19.6 (SD 2.6 years, range 13-25), and 86.9% (374/430) were primarily identified as heterosexual. The demographics of participants sending messages mirrored those of the overall clinic.

All participants sent 5 messages, with 18% (18/100) sending more than the required 5. In the brief posttexting interview, most participants reported no concerns, with 55% (55/100) sending the text message, 18% (18/100) expressing worry about their friends’ response, and 27% (27/100) expressing concern that their friend would not heed their advice (eg, friend would not call the clinic or a friend would not listen). In the 1-week postmessaging interview, no youth reported adverse events or negative outcomes from sending the message.

### Thematic Findings

We identified 4 overarching themes that emerged from participants’ text messages. Nearly all text messages (101/108, 93.5%) included some combination of 2 or more of these themes. The four themes were (1) calls to action, (2) personalized messages, (3) clinic information, and (4) self-disclosure of services received at the clinic or positive experiences and perceptions about clinic services and staff.

### Calls to Action

The most common characteristic of text messages (94/108, 87% of all messages) were calls to action (CTA) that were intended to encourage friends to get tested and know their status, or to get some other service (eg, birth control, a mom’s group, or job services). Some CTAs were more direct than others. For example, some texts directly called for their friend to come to the clinic to receive a service (eg, “…you should check it out and get tested for stds…” (participant 40). Others were less direct, which we coined passive CTA, and included messages that were more general about the importance of staying on top of one’s health (eg, “Its a good idea to come to 3rd st youth clinic and get checked out” (participant 70).

### Personalized Messages

Although clinic staff provided participants with an SMS text message guide including prewritten text messages, most SMS text messages (87/108, 80.6%) were written by participants. Most of these SMS text messages simply informed the recipient of general clinic information and encouraged clinic attendance, while a small number of these messages (n=10) were more personal in nature. For example, some referred to something specific about themselves in the message (eg, “…remember I was a health educator…” participant 102) or referenced something specific to the text recipient (“wen u cum bck frm Texas, [I’m] here at the clinic on 3rd, u shuld stp bi” participant 5). Others started with “hey. This is [name].”

### Clinic Information

More than two-thirds (74/108, 68.5%) of SMS text messages included clinic-specific information. Some included basic information about the clinic, such as the address or other location marker (eg, “…It’s near the new wing stop on 3^rd^…” participant 58), phone number, or hours of operation. Others included specific information about services offered at the clinic (eg, “…They offer free std testing…” participant 78).

### Self-Disclosure and Positive Clinic Experiences

In more than half (60/108, 55.5%) of SMS text messages, adolescents disclosed something about their own clinic participation (34/108, 31.5%) or positive experiences about the clinic (26/108, 24.1%). These included general statements about having been to the clinic, such as “…theres a good clinic ive been going for years…” (participant 48). Others included more specific information on why they attended the clinic: “…I went and got tested. And I know my status…” (participant 26). In addition to disclosing their own clinic attendance, some participants included positive information about their own experience at the clinic (eg, “I just got checked out at the 3rd st clinic, and they were really nice…” (participant 41). Others did not disclose anything about their personal experience but shared positive perceptions of the clinic: “…they will not tell your business to no one so go check them out…” (participant 103). Within this theme, 41.6% (25/60) included both self-disclosure and positive experiences, while most (35/60, 58.3%) disclosed their clinic attendance only or did not disclose receiving any services but shared positive perceptions about the clinic.

### Responses Received from Peers

A total of 40% (40/100) participants reported that they received responses from the friends they texted. Among those reporting that they received a response, most 57.5% (23/40) reported receiving at least 1 positive response, while 10% (4/40) reported that they had received at least 1 negative response. There were some differences in responses depending on the type of message sent. Of those using personalized messages, 32% (28/87) received any response, and of those responses, 60.7% (17/28) were positive. Approximately a quarter (15/100, 15%) of the staff provided SMS text messages as is without making any changes. Of those, 53.3% (8/15) received some type of response, and 37.5% (3/8) of those responses were positive. While a greater proportion of the staff providing SMS text messages received a response compared to the personalized messages, a smaller proportion of them received positive responses. Similarly, of those text messages that used direct calls to action, 32.1% (17/53) received any response, with 70.6% (12/17) of those being positive. Of those using passive calls to action, 41.5% (17/41) received a response, but only 41.2% (7/17) of those were positive responses.

## Discussion

### Principal Results

The overarching purpose of this study was to examine what youths view as motivating and persuasive when participating in peer-driven text messaging approaches to promoting sexual health service access. Consistent with the word-of-mouth communication strategy [12], we found that youths viewed tailoring the content as a facilitator of a response from their intended recipient, as they chose not to use the few predesigned example messages found in the provided guidance. Tailoring messages is a health communication best practice previously used in SMS-based counseling interventions with youths [18-20]. Over 40% (40/100) of participants reported that their peers replied to their message. There were few negative text responses and no negative consequences or adverse events reported by participants in follow-up interviews. This suggests that the messages sent by participants may also be acceptable to the recipients of the message.

We categorized the content of the messages into four themes and noted that most messages used at least two of the four themes: (1) calls to action, (2) personalized messages, (3) clinic information, and (4) self-disclosure of services received or positive clinic experiences and perceptions. Calls to action, where youths encouraged their peers to access HIV or STI testing and other health services, were highly prevalent in the messages that participants crafted. Peer influence has been widely studied in a number of maladaptive behaviors [21]. However, direct peer endorsement of services has been demonstrated to promote health-seeking behaviors of a wide variety, including HIV prevention [22,23], physical activity [24], and most recently, COVID-19 vaccination uptake. Future research should explore the use of peer-designed and peer-delivered text interventions to promote different health-seeking behaviors related to sexual health, including web-based information seeking and the engagement of others in decision-making related to sexual behaviors.

Personalized messages included informing peers about clinic information as well as whether the individual sending the message had accessed the clinic. More than two-thirds of messaging also included specific information about the clinic (eg, location, hours of operation, and services). Informing peers about available services, hours of operation, and location suggests that peers view this information as important for facilitating access. This may be because it facilitated transportation seeking, on which many youths would be dependent [25,26].

Finally, self-disclosure about visiting the clinic and reporting positive experiences may also serve to encourage clinic attendance through reporting patient satisfaction [27]. Self-disclosure may also serve to normalize and destigmatize sexual health service use, which has important implications for future research since stigma is a major barrier to sexual health service use among adolescents and young adults [28,29]. Given that youths often share many similarities with their peers, future research should evaluate the impact of self-reported positive care experiences on health care access and health care stigma experiences among youths.

### Limitations

Several limitations exist with this study’s findings. While we did record the immediate response from message recipients, we were unable to examine if all recipients read the messages or to directly ask recipients their reactions. This study was also conducted within the initial phase of a text message intervention deployed in an adolescent medicine clinic serving youths aged between 12 and 24 years. Therefore, we cannot ascertain the direct relationship between messages and actual testing rates, HIV or STI diagnose rates within this study’s analyses. Further, the patient population included in this study and the larger intervention are likely insured with some existing access to care and may not represent youths and young adults experiencing profound marginalization and the greatest HIV or STI inequities. Lastly, our findings are supported by existing literature but may not have generalizability to all youths, youths in the region where the research was conducted, or the youths included in the larger study.

### Conclusions

The disproportionately high STI and HIV incidence among adolescents and young adults underscores the need for identifying approaches to interrupt the spread of STIs and HIV and prevent new infections, particularly among minority youths. Adolescents rely on text messaging as a primary method of communication, which has implications for leveraging peer influence for health promotion, especially among youths of color who experience the highest HIV and STI incidence. Future interventions should incorporate guidance for youths as they craft messages for sharing with peers. Self-disclosure of care seeking was a common method used by youths, which may be a powerful approach for destigmatizing HIV and STI testing and should be expanded on in future research and trainings with youths as they craft messages. The use of low-cost, easily accessible peer-driven text-based interventions to promote sexual health care service usage among adolescents and young adults has great potential for reducing inequities and improving health outcomes.

## Abbreviations

CTA: call to action
STI: sexually transmitted infection

## Data Availability

The data sets generated during and analyzed during this study are available from the corresponding author on reasonable request.

## References

[1] Centers for Disease Control Prevention (2018). Diagnoses of HIV infection among adolescents and young adults in the United States and 6 dependent areas, 2012–2017. HIV Surveill Suppl Rep.

[2] Cuffe KM, Newton-Levinson A, Gift TL, McFarlane M, Leichliter JS (2016). Sexually transmitted infection testing among adolescents and young adults in the United States. J Adolesc Health.

[3] Zanoni BC, Mayer KH (2014). The adolescent and young adult HIV cascade of care in the United States: exaggerated health disparities. AIDS Patient Care STDS.

[4] Kann L, McManus T, Harris WA, Shanklin SL, Flint KH, Queen B, Lowry R, Chyen D, Whittle L, Thornton J, Lim C, Bradford D, Yamakawa Y, Leon M, Brener N, Ethier KA (2018). Youth risk behavior surveillance - United States, 2017. MMWR Surveill Summ.

[5] Schnall R, Rojas M, Travers J (2015). Understanding HIV testing behaviors of minority adolescents: a health behavior model analysis. J Assoc Nurses AIDS Care.

[6] Anderson M, Jiang J (2018). Teens, social media and technology 2018. Pew Research Center.

[7] Hall AK, Cole-Lewis H, Bernhardt JM (2015). Mobile text messaging for health: a systematic review of reviews. Annu Rev Public Health.

[8] O'Malley TL, Horowitz KR, Garth J, Mair C, Burke JG (2017). A technology-based peer education intervention: results from a sexual health textline feasibility study. Am J Sex Educ.

[9] Wright E, Fortune T, Juzang I, Bull S (2011). Text messaging for HIV prevention with young Black men: formative research and campaign development. AIDS Care.

[10] Perry RCW, Kayekjian KC, Braun RA, Cantu M, Sheoran B, Chung PJ (2012). Adolescents' perspectives on the use of a text messaging service for preventive sexual health promotion. J Adolesc Health.

[11] Social Network Strategy (SNS) for HIV testing recruitment. Effective interventions. Centers for Disease Control Prevention.

[12] Hennig-Thurau T, Walsh G, Walsh G (2014). Electronic word-of-mouth: motives for and consequences of reading customer articulations on the internet. Int J Electron Commer.

[13] Rogers EM, Singhal A, Quinlan MM (2014). Diffusion of Innovations.

[14] Bansal HS, Voyer PA (2016). Word-of-mouth processes within a services purchase decision context. J Serv Res.

[15] Lightfoot M, Jackson-Morgan J, Pollack L, Bennett A (2022). Acceptability and feasibility of peer-to-peer text messaging among adolescents to increase clinic visits and sexually transmitted infection testing: interrupted times-series analysis. JMIR Form Res.

[16] Braun V, Clarke V (2006). Using thematic analysis in psychology. Qual Res Psychol.

[17] Braun V, Clarke V (2014). What can "thematic analysis" offer health and wellbeing researchers?. Int J Qual Stud Health Well-being.

[18] Cortese J, Lustria MLA (2012). Can tailoring increase elaboration of health messages delivered via an adaptive educational site on adolescent sexual health and decision making?. J Am Soc Inf Sci Tec.

[19] Castro RP, Haug S, Debelak R, Jakob R, Kowatsch T, Schaub MP (2022). Engagement with a mobile phone-based life skills intervention for adolescents and its association with participant characteristics and outcomes: tree-based analysis. J Med Internet Res.

[20] Schwebel FJ, Larimer ME (2020). Text message reminders as an adjunct to a substance use intervention for adolescents and young adults: pilot feasibility and acceptability findings. Digit Health.

[21] Henneberger AK, Mushonga DR, Preston AM (2020). Peer influence and adolescent substance use: a systematic review of dynamic social network research. Adolescent Res Rev.

[22] He J, Wang Y, Du Z, Liao J, He N, Hao Y (2020). Peer education for HIV prevention among high-risk groups: a systematic review and meta-analysis. BMC Infect Dis.

[23] Sodik MA (2018). Analysis of improved attitude of youth in HIV/AIDS prevention through the provision of health education with peer education. 2nd Jt Int Conf.

[24] Haidar A, Ranjit N, Archer N, Hoelscher DM (2019). Parental and peer social support is associated with healthier physical activity behaviors in adolescents: a cross-sectional analysis of Texas School Physical Activity and Nutrition (TX SPAN) data. BMC Public Health.

[25] Doll M, Fortenberry JD, Roseland D, McAuliff K, Wilson CM, Boyer CB (2018). Linking HIV-negative youth to prevention services in 12 U.S. cities: barriers and facilitators to implementing the HIV prevention continuum. J Adolesc Health.

[26] Philbin MM, Tanner AE, Duval A, Ellen J, Kapogiannis B, Fortenberry JD (2014). Linking HIV-positive adolescents to care in 15 different clinics across the United States: creating solutions to address structural barriers for linkage to care. AIDS Care.

[27] Wilson KS, Beima-Sofie KM, Moraa H, Wagner AD, Mugo C, Mutiti PM, Wamalwa D, Bukusi D, John-Stewart GC, Slyker JA, Kohler PK, O'Malley G (2017). "At our age, we would like to do things the way we want:" a qualitative study of adolescent HIV testing services in Kenya. AIDS.

[28] Tang W, Ritchwood TD, Wu D, Ong JJ, Wei C, Iwelunmor J, Tucker JD (2019). Crowdsourcing to improve HIV and sexual health outcomes: a scoping review. Curr HIV/AIDS Rep.

[29] Ibitoye M, Lappen H, Freeman R, Jordan AE, Aronson ID (2021). Technology-based interventions to increase point-of-care HIV testing and linkage to care among youth in the US: a systematic review. AIDS Behav.

